# A practical cyberattack contingency plan for radiation oncology

**DOI:** 10.1002/acm2.12886

**Published:** 2020-04-24

**Authors:** Baoshe Zhang, Shifeng Chen, Elizabeth Nichols, Warren D’Souza, Karl Prado, Byongyong Yi

**Affiliations:** ^1^ Department of Radiation Oncology University of Maryland School of Medicine Baltimore MD USA

**Keywords:** business continuity plan, contingency plan for radiation oncology, patient data security, radiation oncology information system

## Abstract

**Purpose:**

This article presents a solution for continuing radiation therapy without interruption in the event of a cyberattack to the radiation oncology information systems (ROIS). This process could be easily deployed to any radiation oncology practice, with little clinical overhead or burden.

**Methods and Materials:**

The solution automatically retrieves all essential information from the clinical ROIS for each under‐treatment patient and periodically (e.g., daily) saves these data to a dedicated secure server for recovery. In the event that the clinical ROIS is not functioning as a result of a cyberattack, this essential information is used to build a new secondary ROIS server to continue radiotherapy treatments until the main ROIS is recovered. Once the cyberattack threat is cleared, the clinical ROIS server is rebuilt from the institution’s enterprise backup. The newly accumulated treatment information for each patient is then exported from the secondary ROIS to bring the clinical ROIS up to date.

**Results:**

The Department of Radiation Oncology at the University of Maryland Medical System implemented this solution for clinical use with the Varian ARIA ROIS in the management of ~250 daily radiotherapy treatments, inclusive of a proton center. This solution was determined to be a feasible and affordable business continuity plan for the radiation oncology practice by minimizing radiation treatment downtime to a couple of hours in a simulated cyberattack drill.

**Conclusions:**

The proposed solution can achieve continuation of radiation therapy treatment without treatment breaks in the event of a cyberattack. It also provides cushion time for radiation oncology departments to rebuild their clinical ROIS systems from the enterprise data backup.

## 1 INTRODUCTION

Cyberattacks are among the major security concerns in the healthcare industry^1^, ^2^ as well as in other industries and government agencies. Cyberattacks on high‐profile companies or government agencies regularly produce significant and disruptive data breaches.^3^ Even high‐tech companies with abundant resources and expertise in cybersecurity fall victim to such attacks. Cyberattacks can result in important data loss, business disruption, financial expenses for restoring system and files, and reputation damage.^4^

In the era of big data, all healthcare information in a hospital or medical center is located in a data center or data warehouse and can be accessed through either the Internet or an intranet service from anywhere within the enterprise, which greatly facilitates patient care. However, such convenience also brings risks. Healthcare providers, such as hospitals, maintain personal information and private health information about patients. Hackers can use stolen or leaked information to benefit financially, damage the institution’s reputation, and, in some cases, to reap “ransom” dollars for restoration of data and systems integrity. As a result, healthcare providers are major targets of cyberattacks.^5^ In 2015, more than 112 million healthcare records were affected by data breaches reported to the Office of Civil Rights under Health and Human Services in the USA.^6^ Each breach costs an average of more than $2 million dollars. Data breaches in health care could even lead to death or personal harm. Researchers in Israel demonstrated that a cyber‐attacker might make use of deep learning to alter CT images, which could result in wrong diagnoses or treatments.^7^

As a result of continuing counter‐efforts from industry, networks have evolved to become more secure. No matter how powerful the antivirus software or how vigilant the employees, however, cyberattacks still succeed in penetrating security barriers and compromising information systems. Cyberattacks are unlikely to stop in the foreseeable future and cannot be fully prevented.

Such attacks, including ransomware and malware, can happen at any time to a radiation oncology department or facility. For most radiation oncology departments, the radiation oncology information system (ROIS) includes all radiotherapy and electronic medical record (EMR) data. When the ROIS is down for any reason, including a cyberattack, the radiation oncology department may be unable to perform any patient‐related treatments and thereby be effectively paralyzed. Radiation treatments are time‐dependent, and such delays could put patient welfare at risk. Therefore, it is imperative for members of the radiation oncology community to have business continuity plans in place, just as in any other industry^8^, ^9^ so that even in the event of cyberattack patient treatment can be continued without interruption.

Current solutions for providing data redundancy for ROIS include enterprise data backup systems and high‐availability rapid recovery protection (HARRP) solutions.^10^ None provide an effective business continuity plan for the radiation oncology community. Because of the intensive data demands of radiation oncology, it would require significant time to restore patient data from the institutional backup system and then check data integrity. The time to bring the ROIS back to operation could be undefined, from 2 days to weeks or months. During such a lapse, radiation treatment would be halted by the unavailability/unreadiness of the ROIS and potential data loss. For ransomware cyberattacks, even if an individual clinic is willing to pay the price to regain data access, time would be required to verify patient data integrity. The US Department of Health and Human Services urges healthcare providers to have a contingency plan to restore daily operations as quickly as possible after compromise from a cyberattack.^11^ The solution outlined here is designed to ensure the continuation of radiation therapy without delay in the event of a cyberattack.

## 2 MATERIALS AND METHODS

The proposed business continuity solution is designed for the Varian ARIA (Varian Medical Systems; Palo Alto, CA) ROIS. However, the underlying approach can be applied to any other ROIS.

### 2.A Prerequisites: hardware configurations

Two computers per each clinical site are needed: a computer to retrieve and store the essential patient treatment information (called the secure data computer, SDC) and a computer with the ROIS installed to serve the function of the ROIS server (called the secondary ROIS computer, SRC). The SDC is a customized bare‐bone Linux computer with a minimum set of installed software. It has the most restrictive access control and enhanced security policies. All network services except for necessary services (such as DICOM service, email service) are disabled to avoid any possible network loopholes for hackers to exploit. Network connectivity is only activated during a short period of time for data retrieval from the clinical ROIS server. At any other time, the computer will remain offline and be isolated from any network connection. It is also physically secured and remote‐logon disabled, with its file system encrypted. The attached mobile USB disks for backup are also password protected. These features make the system much more difficult to hack.

The SRC is used to rebuild a new ROIS. It is a fully functional ROIS system configured by the vendor, with all the necessary information, including treatment machine configurations, beam data and models, template files, etc. The beam data and models are used to verify whether two treatment machines are dosimetrically equivalent so that treatment plans can be transferred between these two treatment machines, especially, for proton treatments. The only difference between the clinical ROIS and this secondary ROIS is that the SRC does not have any patient‐specific information. The SRC is powered off and secured in a safe place at the treatment site until the contingency plan is activated.

### 2.B Daily operation

At 2:00 am every day, the SDC automatically enables its network drive to stay online.

#### 2.B.1 List of under‐treatment patients

**Fig. 1:**
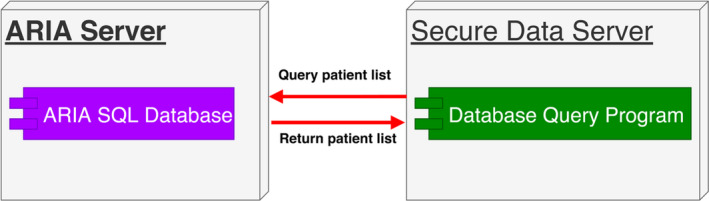
How the under‐treatment patient list is obtained.

Once the network is enabled, the system acquires the under‐treatment patient list by querying the ARIA SQL database (Fig. 1). The two groups of under‐treatment patients include (1) any patient who was treated during the last 3 days and has uncompleted treatment plans and (2) any patient who has not started treatment yet but has one or more treatment‐approval plans scheduled for treatment.

#### 2.B.2 DICOM retrieval

**Fig. 2:**
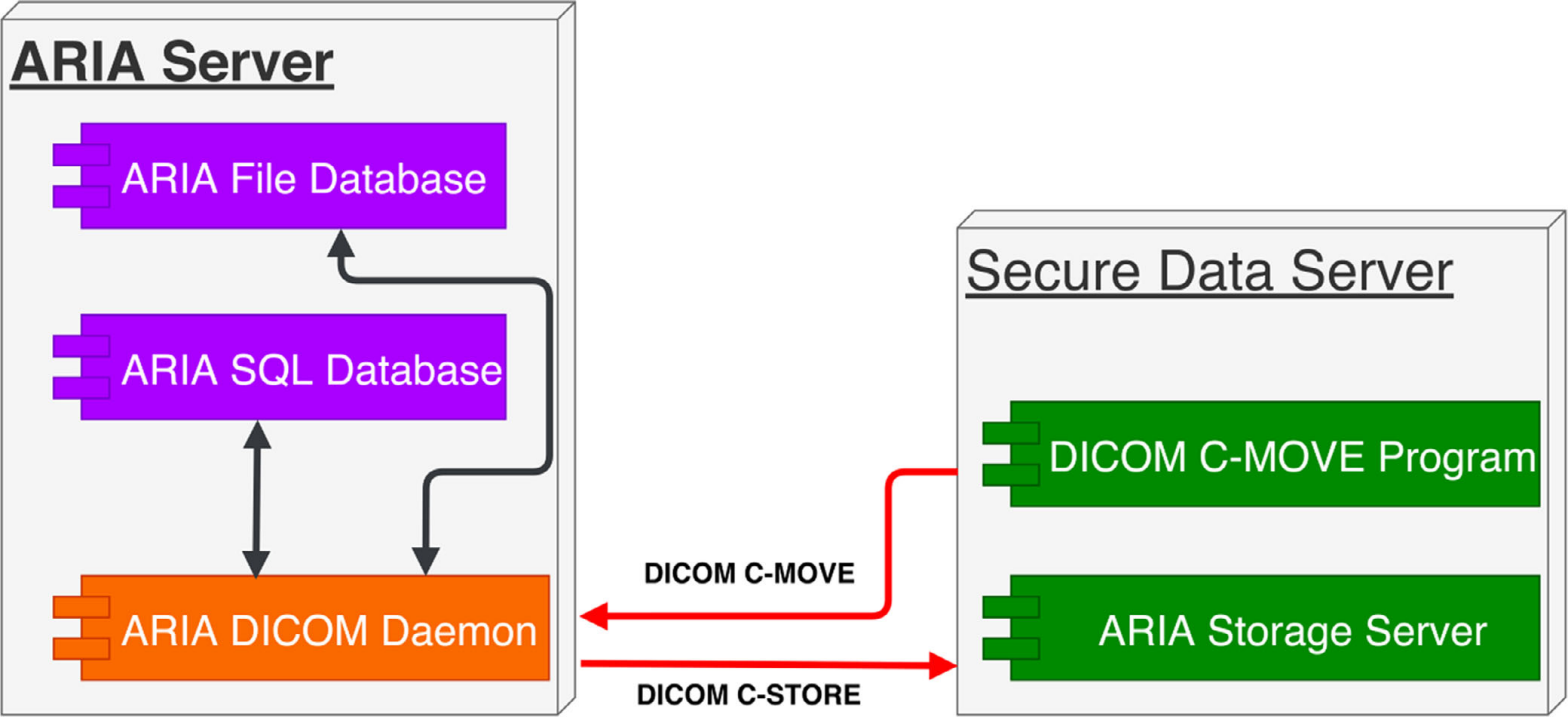
Automatic DICOM file retrieval.

Once the list of under‐treatment patients is retrieved, the system retrieves all the DICOM information for each patient on the list (Fig. 2). This information includes all simulation images and setup images, treatment plans, structures, and treatment records. The system sends a DICOM request to the ARIA DICOM Daemon. The ARIA DICOM Daemon sends the information in DICOM format to the DICOM storage service in the secure data computer. Each day, the DICOM storage service saves all the DICOM information of each patient to a new folder specified by a timestamp. For patients whose treatment has started, the system compares the DICOM files of the latest retrieval with those of the first retrieval. DICOM files are paired according to their DICOM instance universal identification (UID) number. The comparison ignores some unessential DICOM elements, such as instance creation date and time. The comparison results are put into the summary report, which is sent to a group of recipients, such as physicists or dosimetrists. If any essential information in the DICOM files changes due to clinical activities or infections, the system encrypts these DICOM files using a private key^12^ and stores them into a network share that can be accessed by the group of physicists or dosimetrists. For any patient whose treatment has not started, these DICOM files are encrypted by a private key and stored into the same network share. The comparison result report is then sent to the group of recipients.

#### 2.B.3 EMR document retrieval

**Fig. 3:**
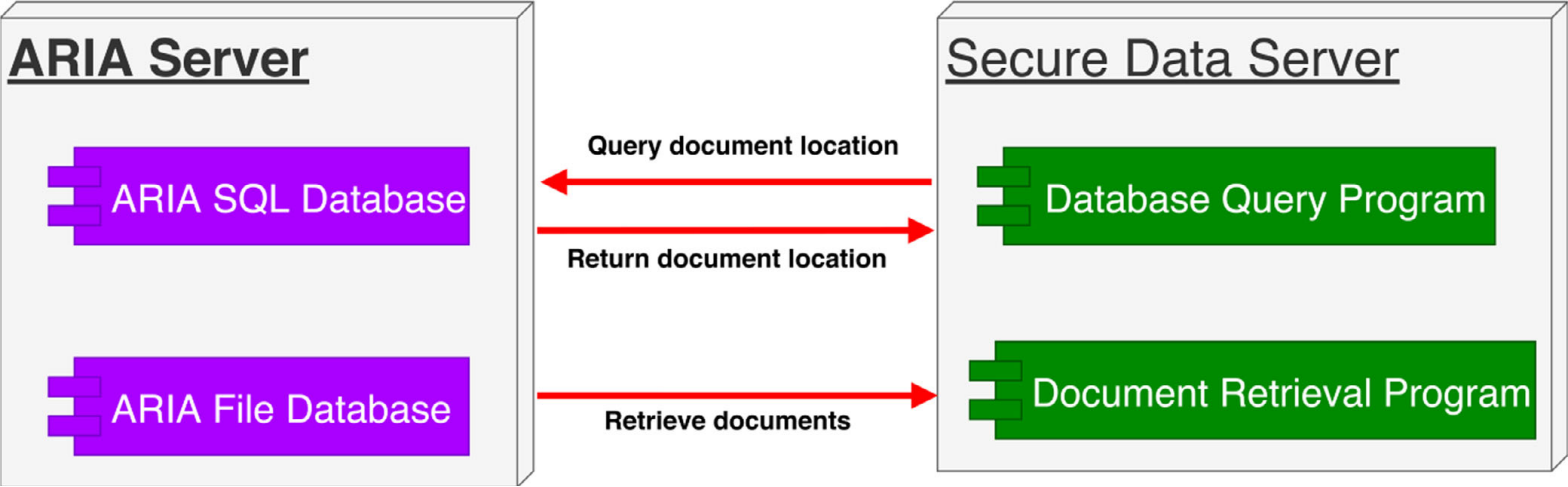
Document retrieval.

For each patient, the essential EMR documents include treatment prescription, treatment plan report, on‐treatment‐visit documents, simulation summary report, etc. First, the system queries the ARIA SQL database server to get the location of each document (Fig. 3). Then, it accesses the ARIA file database to retrieve each EMR document. For a document with multiple versions, only the latest version is retrieved. Then, the system compares the latest retrieval of each document with the first retrieval. The comparison results are put into a report. The report is sent to a group of recipients. If the document has been modified, the modified document is encrypted by a private key and stored into the same network share.

#### 2.B.4 Daily alerts

Each time the system completes retrieval of necessary information, it generates a summary report and then sends this report to designated physicists, dosimetrists, operation managers, IT managers, and/or designated IT staff. Each recipient must keep in mind that if he or she does not receive such daily reports, the system should be investigated immediately. Each day, the group of recipients receives a report about the comparison results through email. If there is any change in any DICOM file or document, each recipient can individually decrypt the encrypted DICOM files or documents using a public key^12^ to save a separate unencrypted copy and then check to determine whether the change is authentic clinically. A suspicious change might indicate a cyberattack, and the entire clinic should be alerted. If the change is clinically authentic, the DICOM files or documents need to be encrypted by the private key of the individual recipient. These are then stored in the same network share identified in “Daily operation” above.

#### 2.B.5 Verification of changes

The system uses the public key of each individual recipient to decrypt the DICOM files and documents that are encrypted by the private key of the recipient. Once any change in any DICOM file or an EMR document is verified by at least two of the recipients, the system updates the first retrieval of these files for future comparison. If such change is not verified within 24 h, the system alerts all the recipients for potential cyberattacks.

Once these operations are completed, the secure data computer disables its network drive and stays offline.

**Fig. 4:**
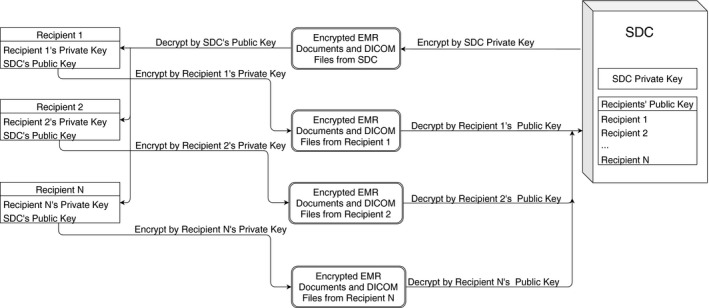
Verification Procedure for DICOM files and EMR documents.

Asymmetric cryptography is used to authenticate the changes of DICOM files and EMR documents. If there are N recipients and one SDC server, there should be (N + 1) public–private key pairs. Each recipient knows her/his private key as well as the SDC’s public key. The SDC server knows its private key and all the N recipients’ public keys (Fig. 4).

### 2.C Hourly operation

From 5:00 am to 9:00 pm daily, the system retrieves daily treatment records of each under‐treatment patient. The purpose of each operation is to prevent loss of the patient treatment records should a cyberattack happen during business hours. Once these operations are completed, the secure data computer disables its network drive and stays offline.

#### 2.C.1 Rebuilding the temporary ROIS after the clinical ROIS is infected

When the contingency plan is triggered due to the clinical ROIS infestation by virus or ransomware, all of the treatment machines will be disconnected from the infested ROIS network and the secondary ROIS computer is set up. All the DICOM files and documents are imported from the SDC to the SRC. The SRC is connected to the treatment machines for radiation treatments. The vendor’s application specialists reconfigure the treatment console for each treatment machine to use the secondary ROIS for treatment.

#### 2.C.2 Restoring patient treatment information back to the clinical ROIS server

Once the clinical ROIS is rebuilt from the enterprise backed‐up data and verified to be ready for back to clinic use, the newly accumulated patient treatment records, acquired kV and cone‐beam CT images, are exported from the secondary ROIS, and all information is imported into the clinical ROIS.

#### 2.C.3 Emergency Scenario

Once the cyberattack is identified and the hospital or department recognizes there is no alternative way to restore radiation therapy treatments within a definite time, the emergency plan will be launched. First of all, the treatment machines will be taken offline. An isolated local network will be used to connect the SDC, the SRC and the treatment control consoles to avoid any further potential cyberattacks. The vender application specialist will create one or multiple ROIS clients (such as Varian ARIA thick clients) on clean computers. Then turn on the SRC and re‐direct the treatment control and the new client computers to the SRC. The local physicists and dosimetrists will import the DICOM files and the EMR documents from the SDC to the SRC one by one. Then the therapists will be able to start the treatments and save the treatment history information back to the SRC. The vendor will work with the local IT staffs to restore the primary clinical ROIS server from the backup system. The physicists and IT staffs will QA the restored patient data in the restored ROIS server. The restoration and data QA might take a couple of weeks for a big cancer center. Once the primary ROIS server is back online, the patient data in the SRC will be exported and imported back to the primary ROIS server. All the patient data in the SRC will be wiped out and taken offline.

## 3 RESULTS

This solution has been successfully implemented and tested in the radiation oncology department at the University of Maryland. The department has a main campus, three community practices, and a proton center. All these radiation oncology practices share a centralized Varian ARIA ROIS server located in the institution’s data center. Each day, about 250 patients receive photon, electron, or proton radiation therapy. Each practice has an SDC and an SRC locally. These are physically secured in each practice. All five SRCs have the same configuration. For daily operation, all five SDCs retrieve all of the under‐treatment patient information, including DICOM files and EMR documents. For hourly operation, each SDC retrieves only treatment records for its own practice.

Each day, it takes about 1.5 h to retrieve all the DICOM files and EMR documents for all of the under‐treatment patients. It takes each practice <5 min to retrieve the treatment records for hourly retrieval operation. It takes about 10 min to export information on each patient from the SDC and import this into the SRC. Another ~10–15 min are required to verify data of each patient in the SRC, which should be done by physicists. Our tests show that the Varian application specialist needs about 1 h to reconfigure each treatment console to use the secondary ROIS server. On average, the clinic should be able to continue patient treatments after about 2 h of downtime. Considering investigation time to triggering the contingency plan and other extra time it may need due to unexpected catastrophic environment, we can resume the treatments within 24 hours from the recognition of the attack.

## 4 DISCUSSION

With cyberattacks posing a threat that continues to grow and cast its shadow over the healthcare industry, having a contingency plan in place for such events is essential.^11^ Usually, the enterprise data backup is considered to be the business continuity plan.^13^ However, several days or more are required for the ROIS vendor and clinic staff to restore backed‐up data and perform all necessary data validation. Although the need for cybersecurity systems in radiation oncology is often discussed,^13^ less attention is given to the importance of a post‐event contingency plan. The solution outlined here is designed to reduce clinic downtime to a couple of hours in the event of a cyberattack. This approach cannot be used as a sole business continuity solution; it must be incorporated into each institution’s enterprise backup for an ultimately disastrous situation. This system provides leeway time so that the radiation oncology department can continue radiation treatments without significant interruption. The system works in an automatic fashion, with no human intervention. Human interventions are needed only when the system requires maintenance or exceptions (such as no daily report) occur.

Varian’s HARRP disaster failover solution^10^ incorporates Double Take® software replication and failover technology (Carbonite, MA) to replicate data in the main ARIA ROIS server to a second ARIA ROIS server at the byte level. In the event of a disaster to the clinical ROIS server, the secondary ROIS server will kick in and resume patient treatments within minutes rather than hours or days as is often cited by vendors. However, this solution is not viable for a contingency plan because the secondary ROIS very likely replicates information from the clinical ROIS server contaminated by virus or ransomware. HARRP was mainly designed for hardware failure and is not immune to virus or ransomware. If the clinical ROIS server is infested by a virus or encrypted by ransomware, the data in the HARRP server might be infested or encrypted as well.

The proposed solution also has the potential to detect a cyberattack before it is identified by other means. The system monitors any changes of DICOM files and EMR documents by comparing DICOM files and EMR documents retrieved daily versus the information retrieved on the first day. When data in the ROIS are tampered with, the exported DICOM files or EMR documents might be altered accordingly. Once these changes are found by comparison, the system alerts a group of designated users for further investigation.

This solution is also cost‐effective. The system should be fully automated and be reporting its daily health. The need for IT support will be minimal. To further reduce the cost, instead of using the secondary ROIS server, the user can load the DICOM RT‐Plan DICOM files directly into the treatment console to treat patients.

Although the contingency plan was implemented and tested for Varian ARIA ROIS, it should be seamlessly ported to other radiation oncology information systems. In case a patient needs to be transferred to another radiation treatment center with either a different type of ROIS or the same type of ROIS, all the retrieved documents and DICOM files will be imperative for helping the patient’s continuing treatments.

## 5 CONCLUSIONS

With help from our ROIS vendor, this system has been fully tested. The test results show that this business continuity plan for radiation oncology is a viable and inexpensive solution to counter cyberattacks against radiation oncology practices. Because of its automatic nature, this solution will add very little burden to clinical workflow.

## CONFLICT OF INTEREST

No Conflict of Interest.
